# When institutions, technologies, and platforms co-evolve: a triadic co-evolutionary framework for public health governance in regulated digital service industries

**DOI:** 10.3389/fpubh.2026.1915580

**Published:** 2026-08-10

**Authors:** Yiding Xiao, Zirui Liao, Hailin Zhang

**Affiliations:** 1 Department of Plastic and Aesthetic Surgery, Peking Union Medical College Hospital, Chinese Academy of Medical Sciences, Beijing, China; 2 Department of Science and Education, Shenzhen Stomatological Hospital, Shenzhen, China

**Keywords:** AIGC, digital health, health equity, health systems, institutional co-evolution, medical esthetics, platform governance, public health governance

## Abstract

How do institutional constraints, technological embeddedness, and platform governance co-evolve to reconfigure the public health governance of regulated digital service industries? This article develops a triadic co-evolutionary framework—the ITP model—that integrates institutional theory, technological evolution theory, and platform ecosystem theory to address this question. The study is positioned as a theory-building, qualitatively oriented single-case analysis supported by secondary documentary evidence and extended through structured cross-national comparison; it does not report primary empirical data. Drawing on China’s medical esthetics social commerce sector as a “theory-laden” extreme case—in which multi-agency public health and consumer-protection regulatory pressure, deep generative-AI (AIGC) penetration, and platforms occupying dual commercial–regulatory roles are simultaneously present—we trace the mechanisms through which the three elements interact via a “constraint–enablement–transmission” channel across three co-evolutionary phases, with explicit attention to the public-health implications of governance dynamics. Cross-national comparative evidence from Republic of Korea and the European Union is incorporated to assess the framework’s conditional generalizability for health-systems governance. The analysis identifies three structurally embedded tensions that the co-evolutionary process appears to generate: a temporal disjuncture between regulatory rigidity and technological elasticity that may compromise public-health responsiveness; a structural contradiction between platform growth imperatives and public-health compliance mandates, expressed as legitimacy deficits; and a compliance-cost escalation dynamic that is consistent with a self-reinforcing feedback loop of concentrated enforcement and market fragmentation, with downstream consequences for the safety, quality, and equitable accessibility of health-related services. We translate these mechanisms into explicit public-health propositions concerning patient safety, health equity, quality of care, health misinformation, and health-system resilience, and we situate the framework against the rapidly expanding 2024–2026 literatures on trustworthy AI, AI governance, algorithmic accountability, and digital-health governance. The ITP framework advances the public-health governance literature by suggesting that these tensions are emergent properties of triadic co-evolutionary interaction—not reducible to any single element’s excess—and by specifying the boundary conditions under which the framework may generalize to other public-health-relevant regulated service industries, including telemedicine, online pharmacy, digital mental health, and platform-based care delivery.

## 1 Introduction

The governance of platform-mediated service industries has become one of the central challenges of contemporary public health governance and regulatory theory. Across a growing range of sectors that directly shape population health—from medical esthetics, telemedicine, and online pharmacy to digital mental health, platform labor markets, and health-related social media—regulators, platforms, technology providers, and public health authorities are engaged in a continuous adaptive process whose systemic properties are poorly understood (1, 2). The present study is motivated by a concrete public-health puzzle: why do well-intentioned regulatory interventions in digitally mediated health-related markets so often produce unintended consequences—reduced service accessibility, displaced but not eliminated unsafe providers, and governed but unaccountable algorithmic decision-making—that ultimately bear on patient safety and population health?

This article proposes a triadic co-evolutionary framework—the “Institutional Constraints–Technology Embeddedness–Platform Governance” (ITP) model—that conceptualizes the co-evolutionary process through which these three elements simultaneously shape and are shaped by one another, with particular attention to implications for public health governance. The framework is developed through an in-depth analysis of China’s medical esthetics social commerce sector, selected as a “theory-laden” extreme case (after Flyvbjerg’s case-logic typology) on the grounds that it exhibits each of the three ITP dimensions in maximal form: regulatory density is exceptional (11 agencies coordinate enforcement across healthcare, pharmaceuticals, advertising, and taxation); technological penetration is unusually deep (AIGC tools, digital-avatar systems, and intelligent recommendation algorithms are central rather than peripheral to service delivery); and the major platforms occupy a structurally distinctive dual role as both commercial intermediaries and quasi-regulatory agents. By examining a case in which co-evolutionary pressures are maximally intense, we aim to identify mechanisms that plausibly operate at lower intensity in a broader class of regulated service industries. The justification for this case logic, and its limits, are developed formally in the Methodology section (Section 3) (3).

The public-health relevance of this inquiry is direct and is foregrounded throughout the article. Medical esthetics is a health-related service in which adverse events—vascular occlusion, infection, esthetic and functional impairment—carry real morbidity, and in which the digital layer (social commerce, AIGC content, algorithmic recommendation) mediates consumer exposure to unsafe procedures and unqualified providers at population scale. The governance dynamics we analyze therefore bear on measurable public-health concerns that are central to Frontiers in Public Health: patient safety and adverse-event monitoring, health equity and access to care, the quality of care, the spread of health misinformation, health-system resilience, and the architecture of digital health governance. We return to these outcomes explicitly in Section 6.

To assess the generalizability of the proposed framework, we incorporate structured cross-national comparative evidence from two additional contexts. Republic of Korea’s medical esthetics market—the world’s most internationally traded in the segment, with a medical-tourism industry generating USD 2.2 billion annually—provides a comparison case with strong institutional differences (single-regulator architecture, export-oriented market logic) and technological similarities (comparable levels of social-commerce penetration and AI adoption). The European Union’s platform-governance framework—formalized through the Digital Services Act (DSA) and the AI Act—provides a second comparison case distinguished by explicit constitutional attention to the dual commercial–regulatory role of platforms, enabling assessment of whether the structural contradictions identified in the Chinese context are jurisdiction-specific or systemic properties of platform governance under regulatory intensification (4, 5).

The article contributes to three bodies of scholarship. For institutional theory, it demonstrates that the co-evolutionary dynamics of platform-mediated industries generate structurally embedded tensions—not smooth regulatory equilibria—and that understanding regulatory outcomes requires attending simultaneously to the technological and platform-governance contexts in which institutions operate. For platform-governance theory, it reveals the accountability deficits that arise when platforms occupy dual commercial–regulatory roles and proposes a typology of governance-adaptation strategies conditioned by the intensity of institutional co-evolutionary coupling. For regulatory theory, including public-health regulatory theory, it identifies the conditions under which concentrated enforcement may trigger self-reinforcing feedback loops—what we term co-evolutionary tightening risk—and proposes circuit-breaker mechanisms designed to interrupt these dynamics. A dedicated subsection (Section 2.5) positions these contributions explicitly against adjacent theoretical approaches, and Section 2.6 situates them against the recent 2024–2026 literature on trustworthy AI, AI governance, algorithmic accountability, and digital-health governance (6).

The remainder of the article proceeds as follows. Section 2 develops the three theoretical pillars and constructs the ITP analytical framework, including a visual conceptual model and an explicit statement of theoretical value added. Section 3 sets out the methodology, case logic, data sources, and analytical procedure. Section 4 presents an empirically grounded analysis of the framework’s three constitutive mechanisms, drawing on longitudinal evidence from the Chinese case and comparative evidence from Republic of Korea and the EU. Section 5 theorizes three structurally embedded tensions and their cross-national variants. Section 6 translates the framework into explicit public-health propositions and population-level implications. Section 7 derives design principles for governance intervention at four levels, with the evidence–prescription link made explicit. Section 8 concludes with theoretical contributions, boundary conditions, limitations, and a research agenda.

## 2 Theoretical foundations and the ITP analytical framework

**Figure 1:**
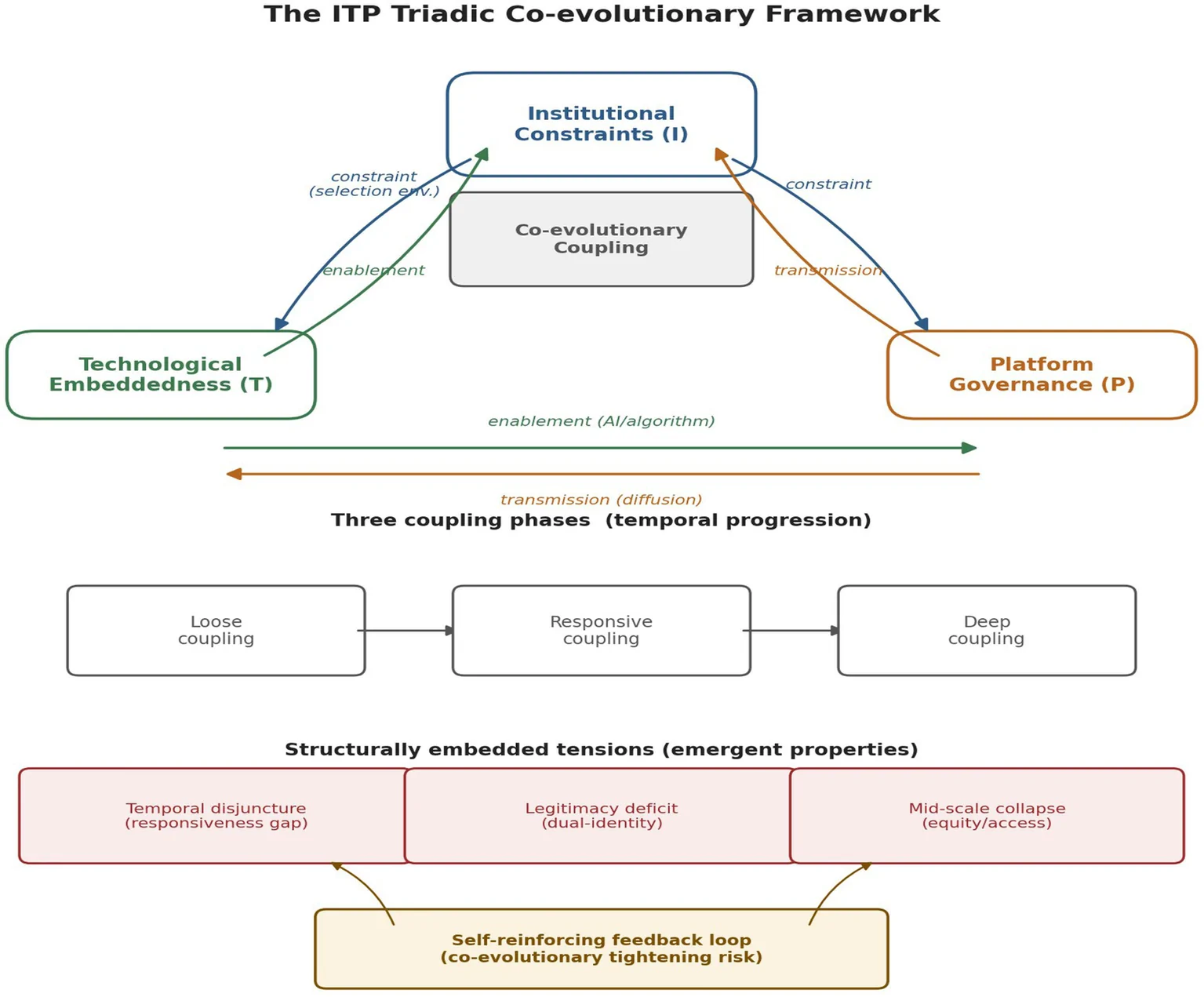
The ITP triadic co-evolutionary framework: elements, channels, phases, and emergent tensions.

This section establishes the three theoretical pillars of the framework and then synthesizes them into the ITP model. A persistent challenge in the co-evolutionary literature is that the conceptual boundaries among “constraint,” “enablement,” and “transmission” are frequently blurred. We therefore define each channel precisely and operationalize it (Table 1) before introducing the visual model (Figure 1).

**Table 1:** The three ITP channels: constraint, enablement, and transmission.

Channel	Direction	Core logic	Empirical signature in the case
Constraint	Institutions → Technology and platform	Coercive and normative limits that delimit which technological capabilities are permitted, required, or penalized, and that set the compliance mandate platforms must operationalize	Eleven-agency enforcement; advertising prohibitions; State Council Order 818 restricting biotechnology to qualified tertiary institutions
Enablement	Technology → Platform and institutions (and feedback)	Instrumental capabilities and evolutionary possibilities that institutions absorb as selection signals and that platforms absorb as AI-moderation, recommendation, and compliance infrastructure	AIGC compliance-content generation; AI skin analysis; platform AI moderation becoming a baseline compliance requirement
Transmission	Platform ↔ Institutions and market actors	The coupling node through which macro-level regulatory requirements are translated into micro-level operational rules and through which technological capabilities reach individual market actors	Xiaohongshu deposit rise (15×); Douyin removal of 77,000 non-compliant products; licensed-practitioner content gating

### 2.1 Institutional theory: rule systems as co-evolutionary agents

Institutional theory provides the foundational vocabulary for understanding how rule systems shape organizational behavior and market structure, including the institutional architecture of public-health governance in platform-mediated industries. Building on North’s distinction between formal and informal institutions, Scott’s three-pillar model distinguishes regulative, normative, and cultural-cognitive dimensions. The regulative pillar encompasses coercive constraints operationalized through laws, licensing systems, and administrative enforcement—in the platform-service context, these include sector access controls, advertising prohibitions, public-health safety standards, and tax-compliance mandates. The normative pillar operates through professional standards, certification requirements, clinical practice guidelines, and industry ethical codes that govern the delivery of health-related services. The cultural-cognitive pillar manifests in how consumers and market actors cognitively differentiate between “compliant” and “non-compliant” providers—a differentiation that progressively reshapes the trust foundations on which health-related service marketing depends and that ultimately influences consumer health-seeking behavior, treatment adherence, and exposure to unsafe practices (1, 2).

Critically, institutional theory has moved beyond treating institutions as static background conditions toward understanding institutional change itself as a dynamic process. DiMaggio and Powell’s analysis of isomorphic pressures demonstrates how coercive, mimetic, and normative forces drive convergence in organizational practices under conditions of institutional uncertainty. North’s transaction-cost framework highlights how institutional change shapes the economic calculus of compliance versus arbitrage. Mahoney and Thelen’s theory of gradual institutional change illuminates how rules are continuously reinterpreted, layered, and converted by actors navigating institutional ambiguity. In the platform-service context, these mechanisms interact with technological change and platform-governance adjustments in ways that existing institutional theory—developed primarily in pre-digital organizational contexts—does not fully capture (7, 8).

### 2.2 Technological evolution theory: paradigm shifts and selective environments

Technological evolution theory attends to the systemic restructuring effects of disruptive technologies on industry configurations. Perez’s techno-economic paradigm theory establishes that the diffusion of general-purpose technologies triggers not merely efficiency improvements but systemic transformations in organizational structures, regulatory frameworks, and sociotechnical environments. Each paradigm creates a “best-practice frontier” that redefines what constitutes appropriate organizational form, generating selection pressures that favor actors capable of mastering the new technological logic. Christensen’s theory of disruptive innovation complements this perspective by tracing how technologies characteristically enter industries from underserved peripheries before progressively displacing incumbent market structures. Nelson and Winter’s evolutionary-economics framework provides the microfoundations: firms routinely search adjacent technological possibilities, with successful variations being selected by both market competition and institutional environments (9–11).

The concept of “selection environment” is particularly important for the present analysis. Institutions do not merely constrain technology adoption; they function as active filters that redirect technological development toward institutionally favored paths, creating what Mokyr terms “useful knowledge” trajectories shaped by the interplay between innovative activity and regulatory recognition. In the AIGC and platform context, this dynamic is unusually transparent: regulatory decisions about which AI applications are permitted, required, or prohibited directly determine which technological competencies are rewarded and which are rendered non-viable, shaping the direction of R&D investment and capability accumulation across the industry (12).

### 2.3 Platform ecosystem theory: governance under dual identity

Platform ecosystem theory provides the analytical apparatus for examining how digital platforms simultaneously create, organize, and govern the markets in which they participate. Rochet and Tirole’s foundational analysis of two-sided markets demonstrates that platform pricing and governance decisions have asymmetric cross-side effects that conventional market analysis fails to capture. Tiwana’s platform-ecosystem framework conceptualizes platforms as architecturally stable cores around which complementary modules are organized by third-party participants, with platform owners simultaneously occupying the roles of “rule-setters,” “value distributors,” and “complaint adjudicators.” Parker et al. extend this perspective by demonstrating that platform governance shapes participant behavior not through direct commands but through the design of interaction rules—a structural form of power that generates significant accountability questions (13–15).

The dual commercial–regulatory role of platforms has received growing attention in the governance literature. Gawer and Cusumano’s work on industry platforms distinguishes between “inner” platform governance (managing complementors) and “outer” platform governance (managing external stakeholders including regulators). Flyverbom and colleagues’ analysis of digital governance highlights how platforms exercise quasi-regulatory power through algorithmic systems that determine visibility, access, and commercial viability for dependent market actors. This quasi-regulatory function creates what Busch terms “private ordering” dynamics: platforms establish and enforce rules that parallel, supplement, and sometimes contradict public regulatory requirements, generating legitimacy deficits when the two systems conflict. When platforms face intensifying institutional pressure—as in the contexts analyzed in this article—the tension between commercial optimization and regulatory compliance becomes a central governance challenge whose resolution strategies have important consequences for market structure, innovation trajectories, and consumer welfare (14–18).

### 2.4 The ITP triadic co-evolutionary framework

Building on these three theoretical pillars, the ITP framework proposes that the evolutionary logic of regulated service industries is a co-evolutionary process in which institutional constraints, technological embeddedness, and platform governance interact via a “constraint–enablement–transmission” mechanism. The three elements are simultaneously autonomous and interdependent: each has its own internal developmental trajectory, yet each is continuously shaped by the others. To keep the conceptual boundaries analytically sharp, we define the three channels as follows (and summarize them in Table 1):

The co-evolutionary interaction operates through these three distinct channels. First, institutional forces supply coercive constraints and normative guidance that directly condition market-actor behavior while indirectly shaping platform-governance strategies and delimiting the applicable boundaries of technology deployment (the constraint channel). Second, technology provides instrumental capabilities and evolutionary possibilities that are shaped by institutional selection environments and absorbed by platforms through investment in AI-moderation systems, algorithmic-recommendation mechanisms, and compliance infrastructure (the enablement channel). Third, platforms serve as the critical coupling node—translating macro-level regulatory requirements into micro-level operational rules (the transmission function) while simultaneously providing the application contexts and diffusion channels through which technological capabilities reach individual market actors (the enablement function, realized through the platform).

The framework incorporates three temporal phases that reflect the progressive tightening of co-evolutionary coupling (summarized in Table 2): a loose-coupling phase (in which the three elements operate with relative independence, constrained only by broadly defined institutional requirements); a responsive-coupling phase (in which tightening institutional conditions trigger reactive technological and platform adaptations, but deep interdependencies have not yet formed); and a deep-coupling phase (in which the three elements become mutually reinforcing, generating a new operational equilibrium characterized by high institutional salience, technology-mediated compliance, and trust-asset construction as the primary competitive logic). This periodization is not merely descriptive; it reflects the directionality of co-evolutionary dynamics—a trajectory from disequilibrium toward systemic coupling that may generate its own endogenous risks when the coupling process overshoots efficient equilibria.

**Table 2:** Three co-evolutionary phases of ITP coupling.

Phase	Institutional salience	Technological role	Platform logic	Empirical marker
Loose coupling	Low/Broadly defined	Optional tool	Traffic maximization; low entry barriers	Permissive content standards; reactive enforcement (pre-2023)
Responsive coupling	Rising/Anticipatory	Compliance infrastructure emerging	Proactive compliance investment ahead of deadlines	2023 eleven-agency joint mechanism; platforms build moderation systems
Deep coupling	High/Systemic	Embedded infrastructure	Legitimacy-seeking; structural reorientation	2025-2026 deposit 15×; 77,000 products removed; 4,231 institutions remediated

The ITP framework makes four theoretical commitments that distinguish it from existing approaches. First, it treats the co-evolutionary process as generative of tensions, not merely of equilibria: the framework is designed to reveal what is suppressed and distorted by co-evolutionary dynamics, not only what is enabled. Second, it incorporates explicit attention to temporal-mismatch effects arising from the differential speeds of institutional change, technological development, and platform-governance adaptation. Third, it positions the platform as the central mediating node rather than a secondary governance instrument, recognizing that the quality and consistency of platform-governance transmission determines whether institutional requirements are effectively operationalized or systematically diluted. Fourth, it is designed with conditional generalizability in mind: the framework applies to regulated service industries simultaneously exhibiting strong multi-agency institutional constraints, deep technological embeddedness in core service processes, and platforms occupying dual commercial–regulatory roles—conditions increasingly common across global service-sector digitalization.

Figure 1 presents the visual conceptual model of the ITP framework, showing the three elements, the constraint–enablement–transmission channels, the three coupling phases, and the three structurally embedded tensions that the co-evolutionary process generates.

### 2.5 Theoretical positioning and value added

The ITP framework is related to, but distinct from, several adjacent approaches. Socio-technical systems theory examines the mutual shaping of technology and social organization but typically treats the regulatory and the platform-governance layers as background rather than as co-equal co-evolving forces. Adaptive governance emphasizes iterative, learning-based regulation but under-theorizes the quasi-regulatory role of platforms. Platform-regulation and algorithmic-governance scholarship analyze platforms as targets of regulation yet rarely model how institutions and technologies co-evolve through the platform node. The regulatory-lag literature identifies timing gaps between innovation and rule-making but does not account for the self-reinforcing feedback dynamics that arise when platforms transmit and amplify institutional constraints. Existing co-evolutionary accounts of technology and institutions tend to be dyadic. Table 3 summarizes what the ITP model adds.

**Table 3:** Theoretical positioning of the ITP model relative to adjacent approaches.

Adjacent approach	Core unit of analysis	What it explains well	What the ITP model adds
Socio-technical systems theory	Technology ↔ Organization	Mutual shaping of artifacts and work	Adds the regulatory and platform-governance layers as co-equal co-evolving forces; specifies public-health outcomes
Adaptive governance	Regulator ↔ Environment	Iterative, learning-based rule-making	Models the platform as a quasi-regulatory transmission node; explains why adaptation alone may not interrupt feedback loops
Platform/Algorithmic governance	Platform ↔ User	Accountability of algorithmic decision-making	Embeds platform governance in a triadic system; shows legitimacy deficits are structurally generated, not merely design failures
Regulatory lag	Institution ↔ Technology (timing)	Speed mismatch between rule and innovation	Generalizes lag into structural temporal disjuncture and links it to compliance-cost escalation and mid-scale collapse
Dyadic co-evolution (tech–institution)	Two elements	Selection-environment dynamics	Third node (platform) as the coupling mechanism that converts dyadic interaction into systemic feedback

### 2.6 Recent advances: trustworthy AI, AI governance, algorithmic accountability, and digital health governance (2024–2026)

The ITP framework directly engages with and extends the fast-growing 2024–2026 scholarship in five key areas. (i) Trustworthy AI: The ITP model operationalizes the normative anchor of trustworthy AI (lawful, ethical, robust) as concrete selection pressures within its constraint channel (19). (ii) AI Governance: The shift from principles to implementation architectures (e.g., EU AI Act, WEF 2024) is treated by the ITP model as institutional selection signals absorbed through the enablement channel. (iii) Algorithmic Accountability: The transparency and appeal mechanisms detailed by scholars like Cheong (20) directly inform the legitimacy-deficit tension and the auditable-governance prescriptions of the ITP framework. (iv) Digital-Health Regulation: The ITP framework provides the meso-level mechanism that connects high-level digital-health governance frameworks (e.g., WHO 2024, NIST AI RMF, OECD 2024) to observable co-evolutionary dynamics in regulated industries. (v) WHO Digital-Health Governance: The WHO’s evolving guidance (2021, 2024) exemplifies the global-scale institutional layer of the ITP’s constraint channel. Synthesizing these streams, the ITP model posits that trustworthy and accountable AI in health are not independently achievable but are jointly conditioned by the triadic co-evolution of institutions, technologies, and platforms. The framework thus serves to operationalize the 2024–2026 agenda by connecting normative and policy objectives to specific, observable mechanisms (19–25).

## 3 Methodology

Both the ambition and the limits of the present study depend on its research design. This section makes the methodological positioning explicit in response to the need for greater transparency about the nature, sources, and analytical procedure of the work. It should be read as a candid account of what the study is—and is not—able to establish.

### 3.1 Research design and epistemological positioning

We position this study as a theory-building, qualitatively oriented single-case analysis supported by secondary documentary evidence, extended through structured cross-national comparison. It is neither a purely conceptual paper nor a primary empirical study reporting newly collected data. More precisely, it is a hybrid: a conceptual framework paper in which the framework is inductively informed by, and iteratively refined against, an empirical record assembled from public documents. We use an abductive logic—moving between the emerging ITP constructs and the documentary evidence—rather than a deductive test of pre-specified hypotheses. This positioning has direct implications for the status of our claims: the empirical material is used to develop, illustrate, and plausibility-check the framework, not to establish causal effects in a strict inferential sense. Throughout the article we therefore use cautious epistemic language (“suggests,” “is consistent with,” “appears to,” “may contribute to”) wherever causal or processual claims are made (26).

### 3.2 Case selection and justification: the extreme/theory-laden case

The Chinese medical-esthetics social commerce sector was selected as an extreme, or “theory-laden,” case (after Flyvbjerg’s case-logic typology). Extreme-case design is appropriate when the research goal is theory-building rather than statistical generalization: by examining a setting in which the phenomenon of interest is present in pronounced form, the analyst can observe mechanisms that may be too weak to detect in more typical settings. The case satisfies the three ITP conditions in maximal combination: (i) exceptional regulatory density (eleven agencies coordinate enforcement across healthcare, pharmaceuticals, advertising, and taxation); (ii) deep technological embeddedness (AIGC, digital avatars, and recommendation algorithms are core to service delivery); and (iii) platforms’ dual commercial–regulatory role. These features make the case unusually “transparent” for observing triadic co-evolution.

We are explicit about the limits of this design. An extreme case maximizes analytic leverage for mechanism identification but constrains the strength of generalizability claims. The intensity of the Chinese configuration means that the absolute magnitude of observed effects (e.g., compliance-cost escalation, market exit) should not be read as representative of lower-intensity contexts. The framework’s broader applicability is therefore stated conditionally and is tested only provisionally through the South Korean and EU comparisons. We return to boundary conditions in Section 8.2.

### 3.3 Data sources

All evidence is drawn from publicly available sources. We categorize them as follows: (a) government and regulator announcements and legal instruments from China, including the National Medical Products Administration (NMPA), the National Healthcare Security Administration (NHSA), the Ministry of Industry and Information Technology (MIIT), the State Administration for Market Regulation (SAMR), the Cyberspace Administration of China (CAC), and the State Taxation Administration; (b) platform-published governance documents—merchant rules, deposit schedules, content policies, and user agreements—from Xiaohongshu (RED) and Douyin; (c) peer-reviewed academic literature on institutional theory, technological evolution, platform governance, and health-service regulation, including the recent 2024–2026 literature reviewed in Section 2.6; and (d) cross-national sources, including the Korean Ministry of Health and Welfare/Korea Health Industry Development Institute and EU legislative instruments (DSA Regulation (EU) 2022/2065; AI Act Regulation (EU) 2024/1689). No new primary dataset was generated, and no human-participant data were collected.

### 3.4 Document selection and framework-guided analytical procedure

Documents were selected through a purposive, theoretically informed protocol rather than exhaustive enumeration. Inclusion criteria were: (i) direct relevance to one of the three ITP elements (institutional change; AIGC/algorithmic technology in service delivery; platform governance rule change); (ii) publication or issuance between 2009 and 2026; and (iii) availability in the public domain in Chinese or English. Exclusion criteria were: promotional or purely commercial material without regulatory content, and items we could not verify against a primary source. We did not pre-register a fixed sample size; document collection was guided by the ITP framework and continued until the public record yielded no further distinct illustration of the three co-evolutionary mechanisms. This is a framework-guided, not a saturation-based, sampling logic.

Our analytical procedure proceeded in three stages, organized around the ITP framework rather than a formal coding scheme. First, the collected documents were read and organized against the three ITP elements and their sub-themes (e.g., “selection environment,” “infrastructuralization,” “dual-identity transmission”); the framework itself served as the analytical template, so no separate codebook was developed. Second, illustrative passages and policy episodes were mapped onto the three transmission channels (constraint, enablement, transmission) and onto the three coupling phases, providing a structured basis for the empirical narrative in Section 4. Third, the framework was refined through abductive iteration: each empirical pattern was checked against the emerging ITP propositions, and the propositions were revised where the evidence did not fit. The cross-national comparison was conducted as a structured focused comparison in which the same ITP template was applied to the Korean and EU materials; observed divergences were used to probe the framework’s conditional claims rather than to confirm a single trajectory. We note explicitly that, because the analysis draws on publicly available documents rather than interview or transcript data, no coded dataset in the grounded-theory sense is produced; the documentary materials remain fully retrievable from the public sources cited in the References.

### 3.5 Evidentiary status of specific quantitative indicators

Several specific quantitative indicators are material to the argument (e.g., the 15-fold increase in Xiaohongshu security deposits, Douyin’s removal of 77,000 non-compliant products, the deregistration or remediation of 4,231 institutions, acquisition costs exceeding CNY 3,000 with commission rates of 60–80%, and a 35% year-on-year rise in customer-acquisition costs). We state their evidentiary status plainly: these figures are drawn from named public sources (platform policy announcements and industry trade reporting) and are used as illustrative indicators of direction and order of magnitude, not as statistically validated measurements. They are subject to well-known limitations—platforms report enforcement counts in ways that serve reputational and regulatory-cooperation purposes; industry cost figures are often estimates; and cross-source comparability is imperfect. We therefore treat them as corroborating signals within a pattern, not as precise effect sizes, and we avoid building causal claims on any single figure.

## 4 Empirical analysis: ITP co-evolutionary mechanisms in action

### 4.1 The institutional constraint mechanism: regulation as a selection environment

The Chinese case offers an unusually detailed empirical record of how institutional constraints function as active selection environments for technological development in a sector with direct public-health salience. The regulatory architecture governing medical esthetics has evolved through multiple generations of escalating complexity, from the 2009 foundational taxonomy to a qualitative shift in 2023 when 11 agencies jointly formalized a coordinated enforcement mechanism that, for the first time, gave market regulators, healthcare authorities, and law-enforcement agencies shared jurisdiction over the same market actors in pursuit of consumer-protection and public-health objectives. The 2026 concentration of interventions—the new VAT Law mandating a 6% rate for for-profit medical institutions, State Council Order No. 818 restricting biotechnology applications to qualified tertiary institutions to safeguard clinical safety, the routinization of “penetrating” corporate-veil-piercing tax enforcement, and the standardization of national pricing categories to enhance price transparency for consumers—represents a case of institutional intervention at a density and scope without precedent in this public-health-relevant sector.

The selection mechanism operates with notable specificity, actively redirecting technological trajectories rather than simply constraining them. Marketing models premised on concepts such as “stem-cell anti-aging” were rendered institutionally non-viable, while AI-based skin analysis and AIGC-assisted compliance-content generation received simultaneous institutional encouragement. This directional selectivity is consistent with Perez’s argument that regulatory frameworks function as “directing mechanisms.” Consequently, this environment gives rise to what Kagan and Scholz term “compliance performance”: when the regulatory regime makes the public demonstration of rule adherence less costly and more rewarding than genuine compliance innovation, actors rationally invest in the former (27).

Cross-national comparison reveals that institutional architecture substantially moderates the directionality of this selection mechanism. Republic of Korea’s single-regulator model (the Ministry of Health and Welfare exercises primary jurisdiction through the Korea Health Industry Development Institute) produces a more coherent selection signal: operators receive consistent guidance on which technologies are approved for which procedures, reducing the compliance uncertainty that generates hedging behavior in multi-regulator environments. The EU’s AI Act, by categorically classifying AI-assisted medical-esthetic diagnostics as “high-risk AI systems” subject to mandatory conformity assessment, produces a third variant: high-barrier technology selection that effectively concentrates AI adoption among large institutional actors while suppressing smaller-scale adoption. These variations suggest that the ITP framework’s “selection environment” mechanism is robust across national contexts, while the specific direction of selection is conditioned by institutional architecture.

### 4.2 The technology embeddedness mechanism: from tool to infrastructure

The technological-embeddedness dimension of the ITP framework concerns the process through which technologies transition from optional tools to foundational infrastructure embedded in the operational logic of both market actors and governance systems, with substantial implications for public-health protection. In the medical-esthetics social-commerce context, this transition has proceeded along five dimensions. Intelligent customer-service systems—powered by large language models fine-tuned on industry-specific corpora—have progressively displaced human consultants for initial inquiry handling, procedure explanation, and post-treatment follow-up, with consequences for the quality of pre-procedure informed consent and post-procedure adverse-event monitoring. Personalized recommendation algorithms leverage multi-dimensional behavioral data to match consumers with procedures and practitioners at a granularity impossible with human curation, raising public-health questions about algorithmic amplification of inappropriate or unsafe procedure demand. AIGC content-generation tools enable compliant promotional-material production at scale, reducing both time costs and compliance risks, but also raising concerns about the verifiability of health claims disseminated through automated content. Private-domain operational systems automate appointment scheduling, follow-up messaging, and loyalty management while generating data assets that support further personalization and that could, in principle, be mobilized for adverse-event surveillance. Digital-advisor avatar systems create scalable expert representations for consumer education and trust formation, with mixed implications for the accuracy of health information conveyed to consumers.

The infrastructuralization of AI has proceeded at institutional as well as commercial levels. By 2026, AI content moderation had become a baseline operational requirement for platform compliance: algorithms identify regulatory violations prior to content publication, effectively translating institutional norms into technical rules embedded in platform architecture. This translation is consequential in ways that parallel the broader algorithmic-accountability literature: when institutional requirements are operationalized as algorithmic rules, they acquire a specificity, consistency, and enforcement velocity that human-administered systems cannot match, but also a rigidity, contextual insensitivity, and opacity that generates accountability challenges existing regulatory frameworks were not designed to address.

Comparative evidence indicates that the infrastructuralization mechanism—though robust across contexts—proceeds at different speeds and along different pathways depending on market structure and regulatory incentives. Republic of Korea’s medical-esthetics market exhibits comparably deep social-commerce penetration (with platforms such as Gangnam Unni and Naver Smart Store serving analogous functions to Xiaohongshu and Douyin), but the infrastructuralization of AI moderation has been slower, reflecting both the single-regulator architecture (which generates less compliance uncertainty) and the smaller domestic market scale (which reduces the ROI of large-scale algorithmic-compliance investment). In the EU context, the DSA’s mandatory algorithmic-transparency and audit requirements for very large online platforms (VLOPs) have driven infrastructuralization along a different pathway: platforms face regulatory requirements to document and audit their moderation systems, generating a form of compliance-driven transparency that differs structurally from the commercially driven infrastructuralization observed in the Chinese case.

### 4.3 The platform transmission mechanism: dual identity and governance adaptation

The platform-transmission mechanism—through which macro-level regulatory requirements are translated into micro-level operational rules governing individual market actors—is the central coupling node of the ITP framework. Its operational logic has evolved through the three co-evolutionary phases in ways that are theoretically instructive. During the loose-coupling phase, the dominant platform logic was traffic maximization: permissive content standards, low barriers to merchant participation, and reactive compliance enforcement characterized a configuration that was commercially rational given the loose institutional environment. In the responsive-coupling phase, platforms began proactively anticipating regulatory developments, investing in compliance infrastructure, and adjusting governance standards ahead of enforcement deadlines. In the deep-coupling phase, platform governance underwent structural reorientation: Xiaohongshu raised security deposits from CNY 20,000 to CNY 300,000 (a 15-fold increase), while Douyin removed 77,000 non-compliant products and restricted certified content categories to licensed practitioners.

These governance adjustments represent deliberate strategic choices to trade short-term revenue for long-term platform legitimacy—choices conditioned by the recognition that regulatory scrutiny had become systemic rather than episodic, and that the public-health stakes of governance failure in health-related service sectors are unusually high. The platform’s dual identity generates a distinctive set of governance challenges with direct public-health implications. As market organizers, platforms must preserve user trust: a single prominent negative-publicity incident—particularly one involving consumer health harm—can erode an institution’s monthly revenue by over 60%, and platform-level trust crises trigger regulatory escalation that increases compliance costs for all market participants while also undermining consumer confidence in the broader digital-health ecosystem. As regulatory conduits, platforms must translate legal requirements, including public-health and consumer-safety requirements, into operationally executable rules—a process requiring both technical infrastructure and interpretive judgment that generates accountability questions when the reasoning behind moderation decisions is not transparent, and that has direct consequences for the speed and consistency with which unsafe practices are identified and removed from circulation.

The EU’s Digital Services Act provides an instructive institutional response to the dual-identity governance challenge. By explicitly designating very large online platforms as “gatekeepers” with affirmative obligations to assess systemic risks and audit algorithmic systems, the DSA attempts to formalize and make accountable the regulatory-conduit function that platforms already exercise informally. Republic of Korea’s approach has been more market-oriented: the Korea Communications Commission has focused on ex ante platform certification rather than ongoing algorithmic oversight, reflecting an institutional preference for entry regulation over behavioral surveillance. These comparative governance architectures suggest that the structural contradiction identified in the ITP framework—between platforms’ commercial and regulatory identities—is a general property of platform governance under institutional intensification, not a China-specific phenomenon, but that jurisdictions have pursued materially different strategies for managing the resulting tensions.

## 5 Discussion: three structural tensions and their theoretical implications

### 5.1 Regulatory rigidity versus technological elasticity: structural temporal disjuncture

The first structural tension generated by the ITP co-evolutionary process concerns the divergent temporal logics of institutional and technological change, with direct consequences for public-health responsiveness. This is not simply an implementation gap of the type familiar from the regulatory-lag literature; it is a structural property of co-evolutionary systems in which the three elements develop at systematically different speeds. Regulatory systems are designed around unified standards, predictable procedures, and risk-minimizing bottom-line logic; technological innovation is inherently predicated on differentiated exploration, iterative experimentation, and productive tolerance for failure. When institutional intensification and technological acceleration occur simultaneously—as the 2026 regulatory concentration in the Chinese med-esthetics case exemplifies—the resulting temporal disjuncture appears to generate perverse compliance dynamics on both sides: regulatory objectives are undermined by technology evolution that outpaces regulatory categories, while technological development is suppressed by regulatory frameworks that cannot distinguish beneficial from harmful innovation.

The concept of “compliance performance” is analytically useful here: when genuine compliance innovation is more costly than the public demonstration of rule adherence, actors rationally invest in the latter rather than the former. This dynamic has been documented in the governance of fintech across multiple jurisdictions, in online-pharmacy regulation in the EU and United States, and in AI-assisted medical-diagnosis governance in both China and the United Kingdom. The consistency of this finding across national contexts suggests it is a structural property of regulatory systems operating in technologically dynamic environments, rather than a jurisdiction-specific pathology. The practical implication—that adaptive governance mechanisms capable of evolving with technology are preferable to stable unified standards in dynamic technological environments—is well-established in the regulatory-theory literature, but its operationalization remains contested across national regulatory cultures (28).

Republic of Korea’s single-regulator architecture produces a structurally different variant of this tension. With the Ministry of Health and Welfare as the primary regulatory authority, Korea has been able to develop technology-specific guidance faster than multi-agency systems, issuing dedicated guidelines for AI-assisted esthetic-consultation tools in 2023. However, the EU experience suggests that even single-stream regulatory architectures face structural temporal disjuncture when the pace of technological change is rapid: the EU’s AI Act, despite extensive consultation, was criticized by industry stakeholders for categorizing AI esthetic tools using risk classifications developed before the generative-AI transition, producing the same mismatch effects in a different institutional form. This cross-national evidence supports the ITP framework’s proposition that temporal disjuncture is a structural consequence of co-evolutionary dynamics, not merely a governance-design failure.

### 5.2 Platform dual identity and legitimacy deficits: a governance-theory problem

The second structural tension concerns the contradiction between platforms’ commercial growth imperatives and their regulatory-compliance mandates—a tension that this article reconceptualizes as a “legitimacy deficit” problem with implications for governance theory. The legitimacy deficit arises because the governance structures platforms develop to manage their dual roles tend toward opacity and inconsistency—opacity because algorithmic-moderation systems are not designed with public accountability in mind, and inconsistency because the commercial and regulatory logics produce contradictory governance signals that platforms resolve through discretionary interpretation rather than principled rule application.

Busch’s analysis of private ordering is directly relevant here: platforms establish and enforce rules that parallel and sometimes contradict public regulatory requirements, generating legitimacy deficits when affected market actors cannot discern whether platform decisions reflect legal requirements, commercial calculations, or algorithmic error. The “rule-legibility problem”—in which inconsistent enforcement criteria across platforms make rational compliance investment extremely difficult—is a specific manifestation of this broader legitimacy deficit. Industry data from the Chinese case indicate a 35% year-on-year increase in online customer-acquisition costs in 2025, a direct consequence of content-restriction policies that reduced organic reach without proportionate quality improvements. The gap between platforms’ formal compliance commitments and the actual experience of affected operators reflects a legitimacy deficit that has practical as well as normative consequences.

The EU’s DSA represents the most developed institutional attempt to address this legitimacy deficit through formal governance design, and it dovetails with the algorithmic-accountability literature. By requiring very large online platforms to publish transparency reports on content moderation, conduct independent audits of recommendation algorithms, and establish accessible appeals mechanisms, the DSA attempts to make the platform’s quasi-regulatory function formally accountable. Early evidence on DSA implementation suggests that these requirements reduce—but do not eliminate—legitimacy deficits: platforms retain substantial discretion in implementing the mandated transparency obligations, and the compliance–commercial contradiction remains structurally present even under enhanced accountability requirements. This evidence supports the ITP framework’s proposition that the structural contradiction between platform growth imperatives and compliance mandates is a fundamental property of the dual-identity configuration, not merely a governance-design problem that can be resolved through better rules.

### 5.3 Co-evolutionary tightening and market diversity: the mid-scale collapse dynamic

The third structural tension concerns the relationship between co-evolutionary tightening—the progressive intensification of institutional constraints, technological-compliance requirements, and platform-governance standards—and market diversity, with significant implications for the equity and accessibility of health-related services. The conventional regulatory-theory expectation is that compliance-cost escalation produces “benign concentration”: marginal-quality operators are displaced while high-quality operators survive and scale. The evidence from the Chinese case is more consistent with a different dynamic that we term “mid-scale collapse”: intermediate-scale operators with genuine quality capabilities but insufficient capital reserves to absorb acute compliance-cost shocks are disproportionately displaced, producing market polarization between large corporate operators with dedicated compliance infrastructure and very small operators below the threshold of regulatory attention, with adverse consequences for geographic and socioeconomic equity in access to safe, quality-assured services.

By March 2025, 4,231 institutions in China’s medical-esthetics sector had been either deregistered or subjected to remediation orders—a 45% year-on-year increase. Per-account acquisition costs exceeded CNY 3,000 in premium segments, with channel commission rates climbing to 60–80%. These indicators are consistent with mid-scale-collapse dynamics: the operators most affected are those with sufficient scale to attract regulatory attention but insufficient resources to absorb the compliance-cost shock. This polarization pattern suppresses the market experimentation and diversity that evolutionary economists identify as the primary driver of quality improvement in service industries, and has public-health consequences including reduced service availability in non-premium segments, weakening of the practitioner pipeline that supplies the next generation of clinicians, and the displacement of services into less regulated channels where consumer protection is weaker.

Comparative evidence from Republic of Korea offers a partial test of this mechanism. Korea’s more coherent institutional architecture and export-oriented market logic have produced a different concentration pattern: foreign-facing clinic clusters in Seoul’s Gangnam district have achieved quality differentiation through international accreditation (the Korean Medical Tourism Association certification system), demonstrating that concentrated markets can be quality-improving when the concentration mechanism is demand-driven rather than compliance-cost-driven. This comparison supports the ITP framework’s proposition that the mid-scale-collapse dynamic is not an inevitable consequence of regulatory tightening per se, but a specific consequence of the multi-agency, compliance-cost-escalation variant of institutional intensification that characterizes the Chinese case.

### 5.4 The self-reinforcing feedback loop: co-evolutionary tightening risk

The three structural tensions interact through the ITP co-evolutionary mechanism to generate a self-reinforcing feedback loop that represents the framework’s most consequential theoretical contribution, with substantial public-health consequences. The loop operates as follows: regulatory rigidity generates temporal disjuncture that inflates compliance costs; rising compliance costs compel platforms to intensify governance to demonstrate regulatory cooperation and manage reputational risk, including the risk of being associated with public-health scandals; intensified platform governance amplifies the effective reach of institutional constraints beyond their formal scope; amplified constraints produce visible market exit that confirms regulatory “effectiveness” while obscuring structural damage to market diversity, service accessibility, and the safety infrastructure that supports consumer protection; and each cycle of tightening reinforces regulatory rationale for continued intensity, closing the loop. From a public-health perspective, the most concerning feature of this loop is that visible market exit is readily misread as evidence of successful enforcement, while the longer-term costs to service accessibility, practitioner diversity, and consumer safety are systematically underestimated.

The theoretical literature provides important analogs. Stigler’s regulatory-capture theory identifies how regulatory concentration generates incumbent-protection dynamics that can become self-reinforcing through political-economy mechanisms. Noll’s analysis of regulatory entry barriers documents how compliance-cost escalation systematically advantages established firms over new entrants, potentially suppressing the entry dynamics that drive industry quality improvement. Frenken and colleagues’ work on platform-economy regulation demonstrates how governance intensity and market structure interact in feedback dynamics that can stabilize at suboptimal equilibria. The ITP framework’s contribution is to show that these feedback dynamics are structurally embedded in the “constraint–enablement–transmission” mechanism—they are emergent properties of triadic co-evolutionary interaction, not merely the byproduct of any single element’s excess. This structural diagnosis has important implications for remediation: element-specific interventions will be insufficient to interrupt a feedback loop sustained by all three elements simultaneously. Effective remediation requires explicitly systemic circuit-breaker mechanisms designed to interrupt the loop at the co-evolutionary system level (29).

Table 4 summarizes the three structural tensions, their cross-national variants, and their public-health consequences; Table 5 maps each tension to the governance response developed in Section 7.

**Table 4:** Three structural tensions and their cross-national variants.

Tension	Core mechanism	China	Republic of Korea	EU	Public-health consequence
Temporal disjuncture	Regulatory rigidity vs. tech elasticity	Multi-agency lag; 2026 concentration	Faster single-regulator guidance	AI act risk classes pre-date GenAI	Reduced responsiveness to safety-relevant innovation
Legitimacy deficit	Commercial vs. regulatory identity	Opacity; 35% CAC-cost rise	Ex ante certification	DSA transparency/audit mandates	Accountability gaps in algorithmic moderation
Mid-scale collapse	Compliance-cost escalation	4,231 institutions; 15 × deposit	Demand-driven quality differentiation	High-risk barrier concentrates AI	Equity/access loss in non-premium segments

**Table 5:** Tensions mapped to governance-design responses.

Tension	Associated governance response (section 7)
Temporal disjuncture	7.1. Adaptive, risk-calibrated institutional architecture (regulatory sandbox; proportionate regulation)
Legitimacy deficit	7.2. Explainable, auditable platform governance; cross-platform harmonization
Mid-scale collapse	7.3. Tiered compliance-cost architecture; public-good compliance infrastructure
Self-reinforcing loop	7.4. Circuit-breaker evaluation mechanisms; incentive-compatible design

## 6 Public health implications: from co-evolutionary mechanisms to population health outcomes

Although the study is documentary and theory-building, its contribution to Frontiers in Public Health depends on articulating how the ITP mechanisms connect to public-health outcomes. We therefore state these links explicitly as propositions—hypotheses grounded in the documentary evidence and in established public-health and AI-in-health governance reasoning—rather than as measured findings. They are offered for subsequent empirical testing (Section 8.4) (30).

### 6.1 Patient safety and adverse-event monitoring

Proposition 1: deeper platform transmission of institutional constraints is associated with faster removal of non-compliant and unsafe providers from visible channels, but the same infrastructuralization of AI moderation may reduce the quality of pre-procedure informed consent and post-procedure adverse-event monitoring when human clinical oversight is substituted by automated systems. The five-dimension infrastructuralization described in Section 4.2 (intelligent customer service, recommendation algorithms, AIGC content, private-domain operations, digital avatars) concentrates both the risk and the surveillance opportunity: the same data assets that personalize marketing could, if governed as a public good, support population-level adverse-event surveillance. The framework thus implies that patient-safety gains from enforcement are conditional on whether platforms are mandated to contribute their data assets to a shared safety-monitoring infrastructure, a point underscored by the WHO’s AI-in-health guidance.

### 6.2 Health equity and access to care

Proposition 2: the mid-scale-collapse dynamic is associated with reduced equity of access. When intermediate-scale, regionally distributed operators exit, service availability in non-premium and non-metropolitan segments contracts, and remaining capacity concentrates in large corporate providers oriented to premium demand. This bears directly on geographic and socioeconomic equity in access to safe, quality-assured services—a core public-health concern. The Korean comparison suggests the direction of this effect is conditional: where concentration is demand-driven and quality-certified, access-equity need not deteriorate. The ITP framework therefore directs equity policy toward preserving the mid-scale segment rather than assuming that any consolidation improves quality.

### 6.3 Quality of care, misinformation, and health-system resilience

Proposition 3: AIGC-enabled content production reduces compliance cost and time but introduces verifiability risks for health claims; algorithmic recommendation may amplify demand for inappropriate or unsafe procedures—classic health-misinformation pathways with population-level effects, and a focal concern of the generative-AI-in-healthcare literature. Proposition 4: health-system resilience is undermined when the self-reinforcing feedback loop displaces the practitioner pipeline that supplies the next generation of clinicians and when services migrate to less-regulated channels where consumer protection is weaker. The framework’s value to public-health practice is therefore not only diagnostic but preventive: it specifies the system-level indicators (operator-exit rate, compliance-cost index, concentration ratio) whose abnormal movement should trigger circuit-breaker assessment before further tightening (21, 31).

## 7 Governance design implications

The governance principles below are derived directly from the empirical mechanisms (Section 4) and the tensions (Section 5) established above. We present them as proposals whose force is conditional on the evidence base; where the empirical link is strong we say so, and where it is suggestive we flag the gap. This section thus addresses the need to make the evidence–prescription link explicit.

### 7.1 Adaptive institutional architecture: from unified standards to risk-calibrated tiers

Interrupting the temporal-disjuncture tension (Section 5.1) implies moving from “one-size-fits-all” compliance frameworks toward adaptive regulatory architectures calibrated to operator scale, risk profile, and compliance history, with explicit attention to the public-health risk profile of the regulated activity. The regulatory-sandbox model, first developed in the United Kingdom financial-services sector and subsequently adopted in Singapore, Australia, and the EU for fintech and health-technology governance, offers a proven institutional template: controlled experimental environments allow regulators to observe risk profiles—including consumer-safety and clinical-risk profiles—before committing to permanent regulatory treatment, enabling institutional frameworks to evolve in pace with technology. The principle of proportionate regulation—under which compliance obligations scale with the scale and risk of the regulated activity, and within health-relevant sectors with the clinical-risk profile of the specific procedures involved—is well-established in EU regulatory design (reflected in the DSA’s differentiated obligations for different platform sizes) and warrants systematic adoption in the multi-agency regulatory architectures characteristic of complex health-related service industries.

### 7.2 Platform governance accountability: toward explainable and auditable systems

Addressing the legitimacy deficit (Section 5.2) requires constructing transparent, auditable moderation frameworks encompassing the complete governance cycle of rule publication, determination, review, appeal, and feedback. The EU DSA’s provisions on algorithmic transparency and independent auditing for very large online platforms, reinforced by the algorithmic-accountability requirements embedded in the EU AI Act and elaborated in the accountability literature, represent the most developed regulatory approach currently in operation: platforms are required to provide human-comprehensible explanations for moderation decisions, maintain accessible appeals mechanisms, and submit to annual independent audits. Generalized adoption of these principles across national regulatory contexts would substantially reduce the rule-legibility problem and the accountability deficits that currently undermine the compliance objectives of algorithmic-governance systems. Cross-platform governance harmonization—achievable through industry-association-led reference frameworks of the type developed by the Global Network Initiative in the human-rights context—would reduce the transaction costs operators bear in navigating divergent rule environments across multiple platforms.

### 7.3 Market diversity preservation: designing against mid-scale collapse

Addressing the concentration–exclusion dynamic (Section 5.3) requires regulatory policy explicitly designed to preserve the mid-scale operator segment that disproportionately bears the compliance-cost shock, with particular attention to the preservation of equitable access to safe services. This suggests a tiered compliance-cost architecture in which the highest-intensity requirements are applied only to high-risk procedures performed by large institutional actors, while proportionate burdens are created for smaller operators in lower-risk activities, with risk calibrated to clinical and public-health criteria rather than purely commercial scale. Industry-association provision of public-good compliance infrastructure—standardized contract templates, shared training resources, automated self-assessment tools, and shared adverse-event reporting systems—can reduce per-unit compliance costs for smaller operators without compromising the regulatory objectives those costs serve and can enhance the systematic collection of safety data that supports population-level public-health surveillance. The Korean model of demand-driven quality differentiation through international accreditation offers an alternative mechanism for quality improvement that does not require compliance-cost escalation to achieve concentration effects and that may be particularly suited to preserving accessibility in lower-tier service segments.

### 7.4 Circuit-breaker mechanisms: systemic intervention against feedback loops

Interrupting the self-reinforcing feedback loop (Section 5.4) requires institutionalized “circuit-breaker” evaluation mechanisms: when regulatory interventions trigger abnormal movements in key market indicators—operator-exit-rate escalation, compliance-cost-index breaches, or concentration ratios exceeding target ranges—staged impact assessments should be automatically triggered before further tightening is authorized. The behavioral-regulatory toolkit offers additional resources: incentive-compatible governance designs that reward compliant behavior through preferential algorithmic treatment, reduced deposit requirements, and enhanced merchant support would reorient operator incentives toward genuine behavioral adaptation rather than compliance performance. Cross-sector learning from analogous regulated industries—online pharmacies, fintech platforms, gig-economy labor platforms—provides empirical reference points for calibrating dynamic-equilibrium regulatory strategies that preserve both market vitality and consumer-protection objectives simultaneously.

## 8 Conclusion

### 8.1 Theoretical contributions

This article has developed and empirically grounded the ITP triadic co-evolutionary framework, integrating institutional theory, technological-evolution theory, and platform-ecosystem theory to model the dynamic mechanisms through which regulated service industries—particularly those of direct public-health salience—undergo governance transformation. Using China’s medical-esthetics social commerce sector as a primary empirical site and incorporating structured cross-national comparative evidence from South Korea and the European Union, we traced the operation of the co-evolutionary mechanism across three phases—from loose coupling through responsive coupling to deep coupling—and identified three structurally embedded tensions generated by the process: temporal disjuncture between regulatory rigidity and technological elasticity that may compromise public-health responsiveness; legitimacy deficits arising from the platform dual-identity contradiction that undermine health-related governance accountability; and mid-scale-collapse dynamics produced by compliance-cost escalation under concentrated enforcement that may erode service accessibility and practitioner diversity.

The framework’s primary theoretical contribution lies in demonstrating that these tensions are emergent properties of triadic co-evolutionary interaction—they cannot be attributed to any single element’s dysfunction and therefore cannot be remediated through element-specific interventions. This structural diagnosis advances the institutional-theory literature by showing that regulatory outcomes in platform-mediated industries depend critically on the technological and platform-governance contexts in which institutions operate; the platform-governance literature by revealing how the dual commercial–regulatory identity generates structural legitimacy deficits that persist even under enhanced formal accountability requirements; and regulatory theory—including public-health regulatory theory—by formalizing the co-evolutionary tightening-risk mechanism through which concentrated enforcement can become self-defeating, with adverse consequences for the very population-health outcomes the regulatory system was designed to protect.

### 8.2 Boundary conditions and scope of generalizability

The framework possesses conditional generalizability to regulated service industries that simultaneously satisfy three criteria: exposure to multi-agency regulatory pressure with high institutional-change frequency; deep technological embeddedness—particularly AIGC and intelligent-recommendation systems—in core service-delivery processes; and platforms occupying dual commercial–regulatory roles. Candidate domains include telemedicine and digital-health consultation platforms, online pharmacy and direct-to-consumer health-product markets, digital mental health service platforms, online medical-advertising ecosystems, and platform-mediated long-term-care services, all of which are increasingly subject to the same triadic co-evolutionary dynamics analyzed here and to the digital-health governance frameworks reviewed in Section 2.6.

We specify where the framework is expected to apply less directly. It is least transferable to (i) physically delivered, low-technology health services with minimal platform mediation; (ii) sectors in which a single regulator and a coherent institutional signal prevail and the multi-agency selection-environment effect is absent; and (iii) contexts where platforms do not exercise quasi-regulatory transmission (e.g., pure payment rails). The direction and intensity of co-evolutionary dynamics are conditioned by three contextual factors: institutional configuration (single vs. multi-agency), market structure (domestic scale and concentration), and regulatory culture (entry-regulation vs. behavioral-surveillance preference). The cross-national evidence incorporated here suggests the core mechanisms—selection environment, infrastructuralization, and dual-identity transmission—operate across different national institutional architectures, while their specific direction and intensity are conditioned by these factors.

### 8.3 Limitations

The study’s limitations are substantial and are stated candidly. First, it relies entirely on documentary evidence; no primary empirical validation (e.g., operator-level or patient-level data) was conducted, so the framework’s claims about public-health outcomes (Section 6) are propositions, not findings. Second, the single extreme-case primary design constrains causal identification and statistical generalizability; the Chinese configuration’s intensity may over-state effect magnitudes for lower-intensity contexts. Third, there is potential contextual bias toward China, partially mitigated but not eliminated by the Korean and EU comparisons, which remain thinner than the primary case. Fourth, the specific quantitative indicators used are drawn from public and industry sources whose measurement standards are not uniform and whose evidentiary status is illustrative rather than definitive (Section 3.5). These limitations are not fatal to a theory-building study but define the evidentiary threshold that future research must cross (26).

### 8.4 Future research

Longitudinal panel-data analyses tracking operator-level responses to co-evolutionary phase transitions would enable formal estimation of co-evolutionary effect magnitudes, including effects on service quality, adverse-event rates, and consumer-health outcomes. Cross-national comparative case studies employing systematic comparison across multiple regulated service industries—including other health-relevant sectors such as telemedicine, online pharmacy, and digital mental health, and engaging the emerging digital-health governance evidence base—would allow more precise specification of the ITP framework’s boundary conditions and the public-health conditions under which it applies. Experimental and quasi-experimental approaches to regulatory-intervention evaluation—exploiting differential enforcement timing across regional jurisdictions—could provide identification strategies for the feedback-loop mechanisms theorized here, including their impact on consumer health and service accessibility. Agent-based modeling approaches could simulate the co-evolutionary dynamics identified in this analysis to generate testable predictions about feedback-loop onset conditions, circuit-breaker effectiveness, and the population-level public-health consequences of alternative governance configurations.

## Data Availability

All data are from publicly available government announcements, platform policy documents, and published academic literature. These sources are listed in the References section. No formal repository was used as no original or aggregated dataset was generated.
